# Supplementary material for: Podocyte specific knockout of the natriuretic peptide clearance receptor is podocyte protective in focal segmental glomerulosclerosis
Source: PLoS One. 2025 Mar 10;20(3):e0319424. doi: 10.1371/journal.pone.0319424 (PMC11892885; doi:10.1371/journal.pone.0319424)
Supplement: S1 Data — (PDF) [file pone.0319424.s001.pdf]

Figure S1

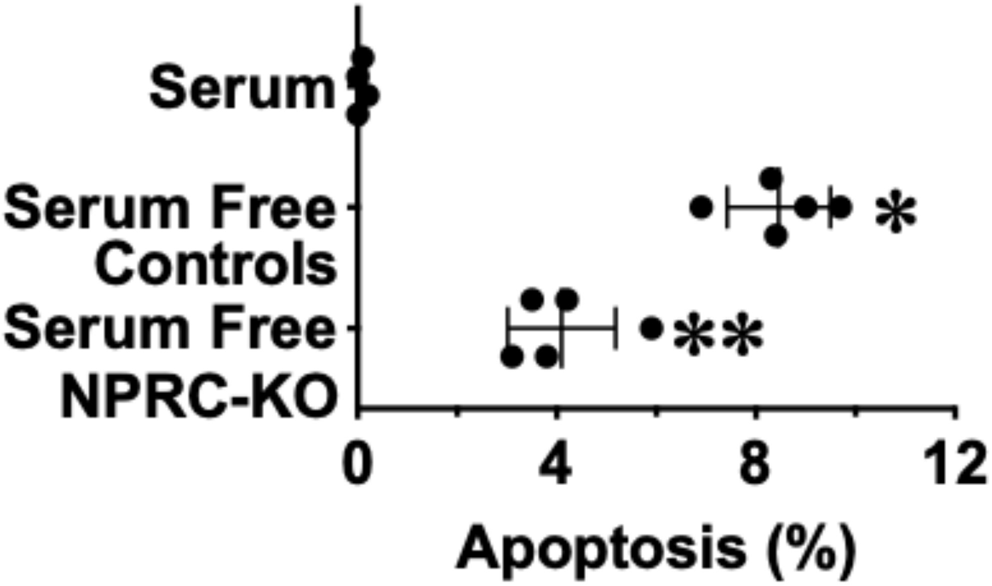

Figure S1: Serum deprivation significantly increased apoptosis in control podocytes and KD of NPRC inhibited the apoptotic response in the absence of exogenous NPs. KD cells and controls were combined in the serum treated group.
* < 0.001 versus serum; ** < 0.001 versus serum free.

Figure S2

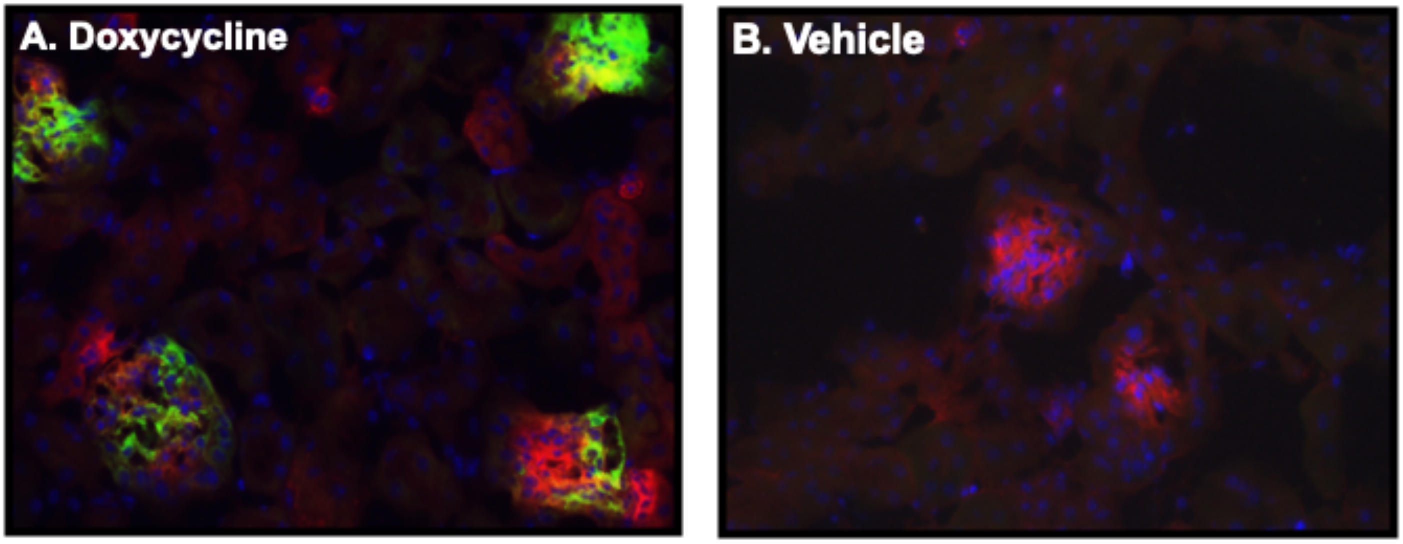

Figure S2: Cre-mediated recombination in vivo. These studies used a transgenic (TG) mouse expressing a reporter allele that contains a cell membrane-localized red fluorescence transgene (td-Tomato) with widespread expression in all tissues and cell types. Expression of Cre recombinase induces a cell membrane-localized green fluorescence protein (EGFP). For the studies, we crossed the tdTomator mice with a TG mouse expressing a doxycycline inducible Cre-recombinase construct. Treatment with doxycycline induces green fluorescence specifically in podocytes, with red fluorescence confined to other cell types (panel A). In contrast, little EGFP expression was observed in mice treated with vehicle (panel B).

Figure S3

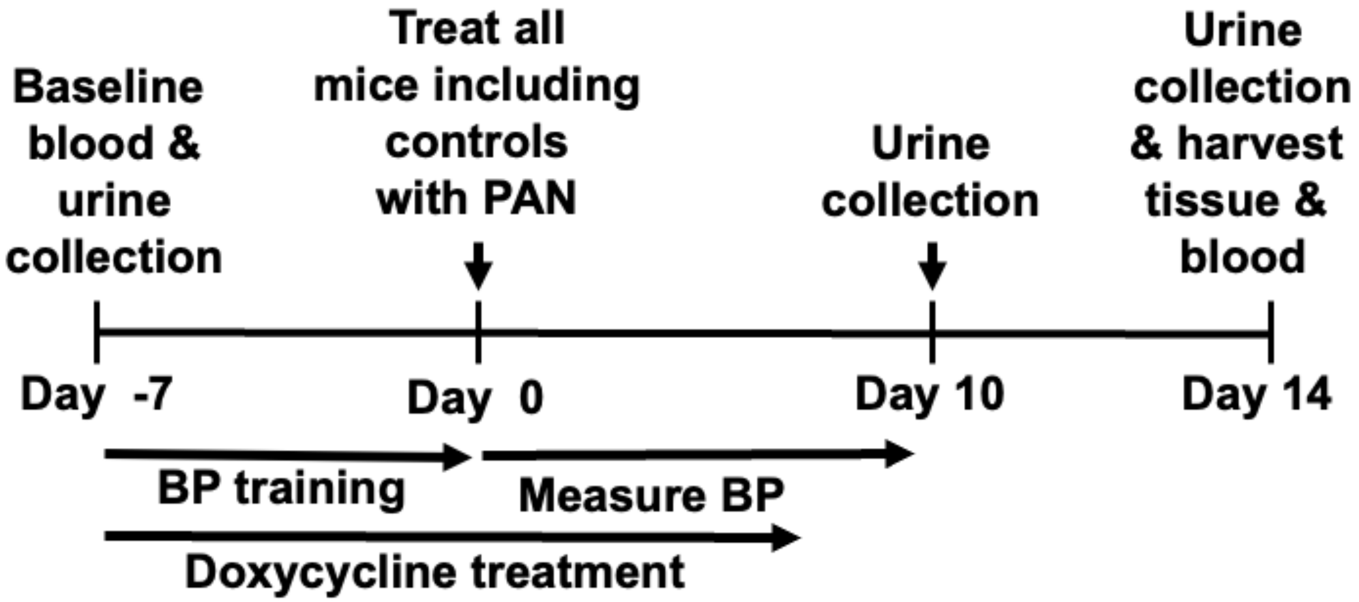

Figure S3: Schematic of the experimental protocol.

Figure S4

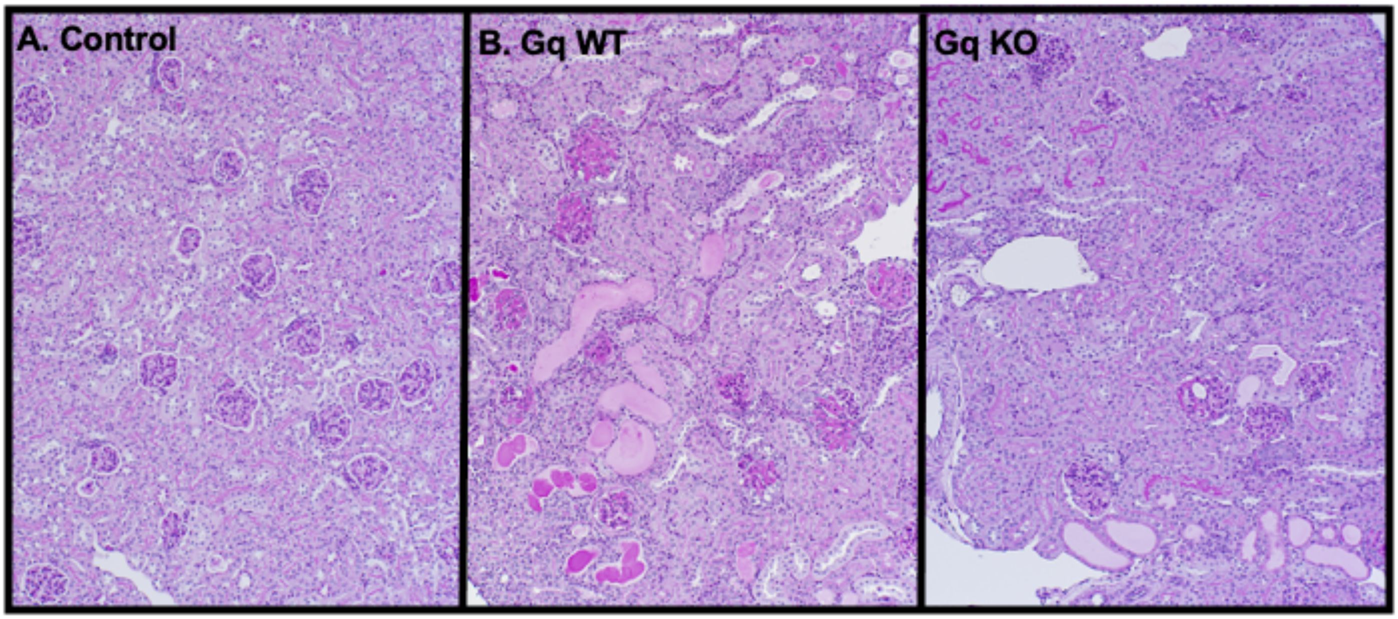

Figure S4. PAS (Periodic acid-Schiff) stained of kidney specimens. A. Low power view of control mice (mice do not express Gq). B. Low power view of Gq mice. C. Low power view of Gq KO mice. NOTE: Periodic acid-Schiff (PAS) stained kidney sections.

Figure S5

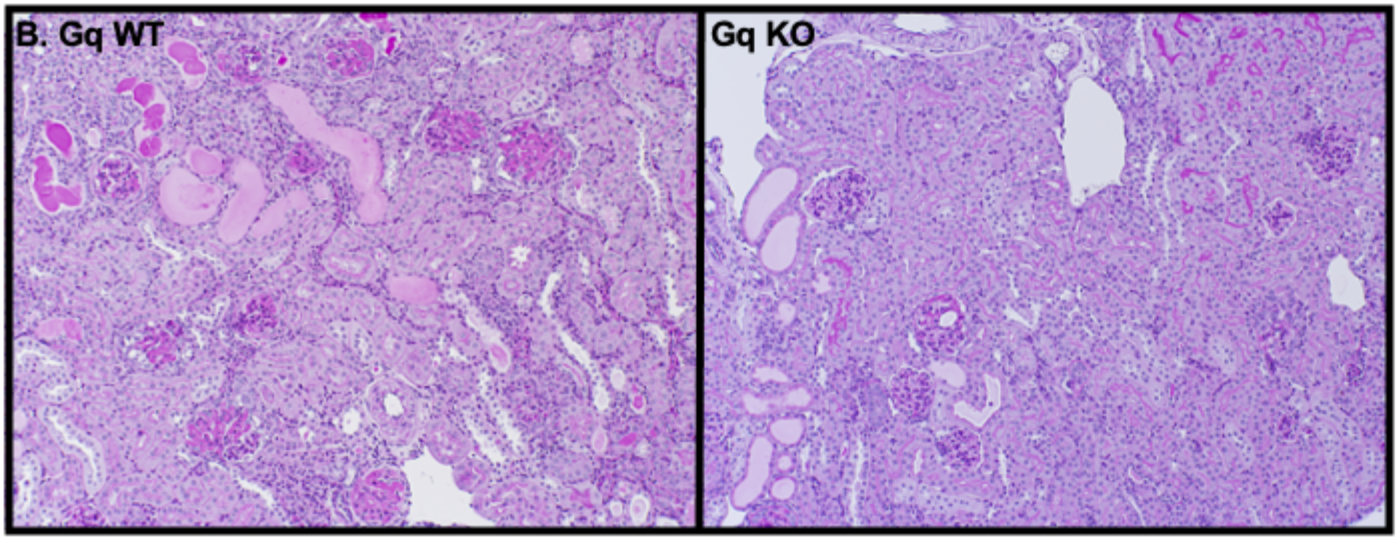

Figure S5. Enlarged views of Gq mice. A. Gq mice. B. Gq KO mice. NOTE: Periodic acid-Schiff (PAS) stained kidney sections.

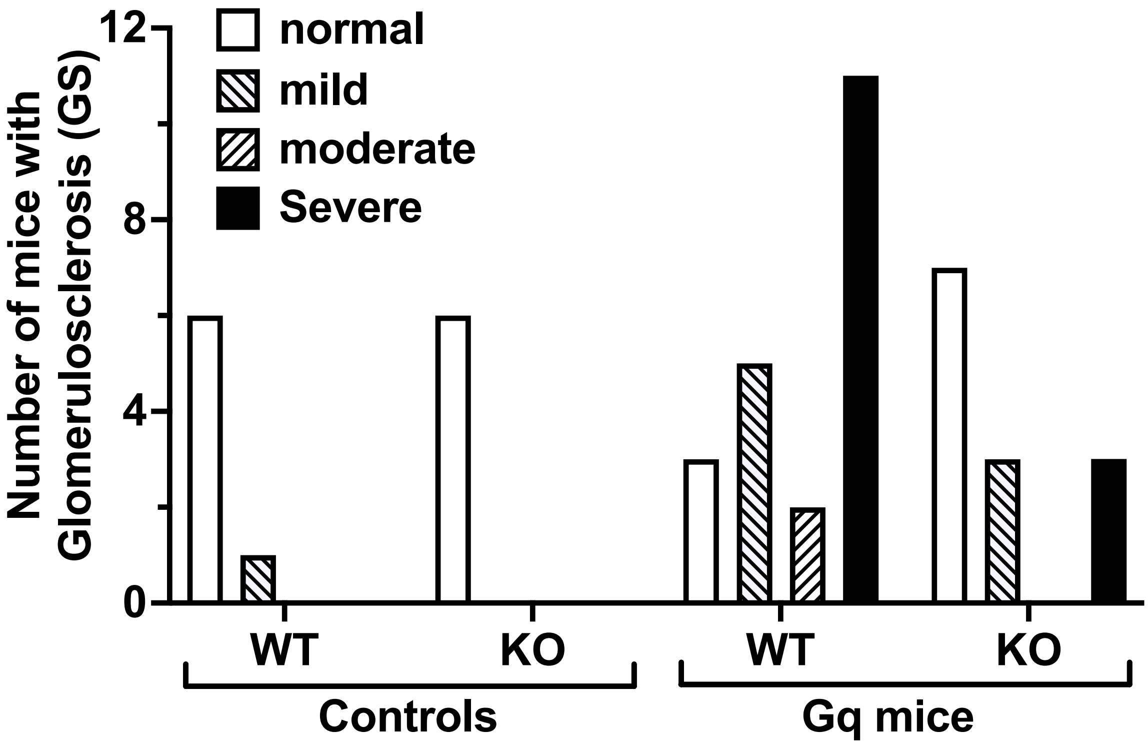

Figure S6. Severity of glomerulosclerosis (GS) in controls and transgenic mice (Gq mice).

Figure S7

Mouse lines:
NPRC-flox/flox: Express 2 "floxed" alleles of NPRC
NPHS2-rtTA : Reverse tetracycline transactivator (rtTA) under the control of the podocyte specific podocin promoter
NPHS2 (NOTE: The protein product of the this transgene permits doxycycline controlled transcriptional activation of the tet operator sequence (tetO)
tetO-Cre: Cre recombinase under the control of tet operator sequence (tetO)
tetO-Gq: Constitutively active Gq alpha subunit [Gq(Q>L)] under the control of tet operator sequence (tetO

The following lines are maintained in our lab:

1. FVB/NJ NPRC-flox/flox mice expressing NPHS2-rtTA and tetO-Gq(Q>L) (Gq mice). These transgenic (TG) mice express a doxycycline inducible, constitutively active Gq-alpha subunit [Gq(Q>L)], which sensitizes mice to the podocyte toxin puromycin aminonucleoside (PAN)
2. FVB/NJ NPRC-flox/flox mice express NPHS2-rtTA and tetO-Cre (Inducible Cre mice). These TG mice express a doxycycline inducible Cre recombinase.

BREEDING STRATEGY

NPRC-flox/flox tetO-Gq (Single transgenic) **X**. NPRC-flox/flox NPHS2-rtTA & tetO-Cre (**Double TG mice**)

OR

NPRC-flox/flox tetO-Cre (Single transgenic) **X** NPRC-flox/flox NPHS2-rtTA & tetO-Gq (**Double TG mice**)

TO CREATE

NPRC-flox/flox NPHS2-rtTA, tetO-Gq & tetO-Cre (**Triple TG mice**)

Triple TG mice: After doxycycline treatment, Gq(Q>L) is expressed and NPRC is deleted specifically in podocytes.

Figure S7. Breeding strategy to create transgenic mice.

Table S1

Table S1. Albuminuria (µg/mg creatinine) in controls (lack Gq)

	Baseline	Day 10	Day 14
Wild type (WT)<br>(N = 6)	59 ± 12	122 ± 28	144 ± 61
Knockout (KO)<br>(N = 6)	58 ± 13	150 ± 42	237 ± 130

N is the number of mice in each group, No statistically significant differences between baseline and day 10 or day 14, or between WT mice and WT-KO mice after correcting for multiple comparisons.

Table S2

Table S2. Quantitative RT-PCR Results

	Controls (ng/dl)	Gq-WT (ng/dl)	GQ-KO (ng/dl)
Wilms Tumor 1 (WT1)	1.0 ± 0.16 (N = 20)	0.99 ± 0.17 (N = 12)	1.87.0 ± 0.36* (N = 12)
Synaptopodin	1.0 ± 0.18 (N = 20)	1.0 ± 1.01 (N = 12)	1.5 ± 0.50 (N = 12)
Podocalyxin	1.0 ± 0.21 (N = 20	0.76 ± 0.16 (N = 12)	1.09 ± 0.28 (N = 12)
Transforming Growth Factor-b (TGFb)	1.0 ± 0.16 (N = 20)	4.8 ± 1.55** (N = 12)	5.25 ± 1.76† (N = 12)
Interleukin 11 (IL-11)	1.0 ± 0.35 N = 20)	2.96 ± 1.10 (N = 12)	2.05 ± 0.84 N = 12

*P < 0.0174, **P = <0.05, †P = <0.025, N is the number of mice
