# Supplementary material for: Podocyte specific knockout of the natriuretic peptide clearance receptor is podocyte protective in focal segmental glomerulosclerosis
Source: PLoS One. 2025 Mar 10;20(3):e0319424. doi: 10.1371/journal.pone.0319424 (PMC11892885; doi:10.1371/journal.pone.0319424)
Supplement: Attachment — Submitted filename: Respomse-to-Reviewers.pdf [file pone.0319424.s002.pdf]

Reviewer #1:

1. Histologic studies: Immunohistochemistry staining is diffuse and lacks negative controls.

We had significant difficulty locating antibodies for the immunohistochemistry studies, and were unable to locate antibodies for mouse kidney. As a result, we attempted to stain human kidney specimens with several antibodies and the current figure was the result. The antibody we used stained multiple cell types in the kidney the kidney consistent with published single cell transcriptomic studies (for example see reference [1]) Little staining was detected in the isotype control antibody panels. We agree that the images a suboptimal and it is also possible that the diffuse staining in the tope panels of Figure 1A could be non-specific. As a result, we are removing Figure 1A from the manuscript.

2. Scale bars are missing:

We have added scale bars to the current histology pictures figures.

3. Alternative considerations: Did not consider that the "protection" of NPRC-deficient podocytes was partly mediated by inhibition of the Gi/Gq-coupling functions of this receptor.

We are aware of alternative signaling pathways linked to NPRC and we discussed this possibility in a previous publication examining the effects of pharmacologic NPRC blockade in a mouse model of FSGS [2]. We apologize for not include this possibility in the current publication and we have amended the manuscript to address this possibility in the revised manuscript.

4. Nomenclature: for many good reasons, the IUPHAR recommended to name NPRA and NPRB as GC-A and GC-B. At least you should clarify this nomenclature.

We have acknowledged the alternate designations for NPRA and NPRB in the text of the manuscript.

5 Introduction: Contradictory sentences: "1. NPs potently stimulated cGMP generation and inhibited podocyte apoptosis in cultured podocytes[17], and 2. Pharmacological blockade of NPs in vivo enhanced urinary cGMP excretion and significantly reduced albuminuria in a mouse model of FSGS[17]." These two sentences obviously contradict each other. Please correct them or discuss appropriately.

We apologize for the error, and we have corrected the mistake as outlined below.
The corrected statement is shown below:

NPs potently stimulated cGMP generation and inhibited podocyte apoptosis in cultured podocytes[17], and 2. Pharmacological blockade of **NPRC** in vivo enhanced urinary cGMP excretion and significantly reduced albuminuria in a mouse model of FSGS[17]." These two sentences obviously contradict each other. Please correct them or discuss appropriately.

6. Figure 1A: The quality of the images in Figure 1A is very low and must be markedly improved.

We agree that the Immunohistochemistry studies are suboptimal. See response #1 above.

6. Figures 1B and C illustrate "statements" (CNP is produced by podocytes and binds to…; NPRC clears CNP from the circulation) which were not experimentally addressed by the present studies. If these statements/schemes are based on published data, then please refer those in the legend. As far as this reviewer knows, nobody has previously shown that "NPRC clears CNP from the circulation").

The natriuretic peptide (NP) clearance receptor (NPRC) binds ANP, BNP, and CNP with a high affinity in the low picomolar range [3-6] and either pharmacologic blockade or KO of NPR-C suggests that a major function of NPRC is to clear NPs from the circulation [7, 8]. The negative regulatory effects of NPRC on the functions of CNP have been demonstrated in bone using mouse models. In this regard, mutations that disrupt the function of genes encoding CNP or NPRB (CNP receptor) causes dwarfism [9-11]. In contrast, disruption of the gene encoding the clearance receptor (NPRC) cause bone overgrowth [8]. These data suggest that: 1. A major function of CNP is binding to NPRB and stimulating bone growth, and 2. the stimulatory effects of CNP on bone growth are augmented by decreased clearance of CNP from the circulation by NPRC.

7. How is the expression of NPRB and NPRC in these cells?

We have provided data on expression of NPRA, NPRB and NPRC in Figure 2. The major findings were: 1. NPRC expression was effectively decreased in KD cells, 2. NPRC is robustly expressed in podocytes compared to either NPRA or NPRB, and 3. NPRB expression is downregulated in NPRC KO mice.

8. **Is it correct that CNP barely increased cGMP contents of cultured control podocytes but raised the cGMP contents of NPR-C KO podocytes by more than 50-fold? This is a surprisingly huge difference. It suggests that in presence of NPR-C, CNP/NPRB/cGMP signaling is almost fully blocked. Why?**

It is correct that CNP induced cGMP generation is significantly blunted by the high expression levels of both NPRC [1, 12, 13] and proteolytic cleavage of NPs by neprilysin [2, 14, 15]. Moreover, the 0.01 nM concentration of CNP is at the lower limit of NPRB binding affinity [5, 6, 16], which severly limits the response to CNP at the 0.01

nM concentration (Figure 1E). As a result, calculating the fold increase in cGMP generation using a number close to zero results in a very large fold change. In contrast, the higher concentrations of CNP are more effectively bound by NPRB (GC-B) and results in more robust cGMP response in, although the response is likely tempered by the by both the clearance receptor (NPRC) and peptide cleavage by neprilysin (both are expressed in podocytes [2]).

9. **Figure 1E: As compared: how are the cGMP responses of control and KD podocytes to , and/or BNP?**

We previously measured cGMP generation using 1μM concentrations of ANP and CMP. In these studies, ANP stimulated cAMP to a greater extent than CNP but the difference was not statistically different [2]. In the current study (Figure 2A), we saw the same pattern. ANP stimulated a greater increase in cGMP generation compared to CNP, but the differences were not statistically different at the 1.0 nM or 10 nM concentrations. In addition, the same pattern was observed in NPRC KD cells, although cGMP response was enhanced compared to cells expressing NPRC. See Figure 2A for current results.

10. Figure 1G: Results are unclear and possibly incomplete. Please show the % apoptosis in control and KO cells in Serum and Serum-free medium. Is there a difference in serum-containing medium between genotypes?

As described in the initial submission, we combined serum treated control and KD podocyte data because little apoptosis was observed in both groups. In the revised manuscript, we have repeated the experiments and separated both the serum and serum free data for KD and control podocytes. In addition, we have increased the duration of the serum deprivation to enhance the apoptotic effect to increase the apoptotic response and, in turn, provide a greater range to enhance sensitivity for detecting differences. In the current study (Figure 1F), KD of NPRC reduced apoptosis induced by serum deprivation, and this beneficial effect of NPRC KD was partially attenuated by pharmacologic blockade of NPRB with pharmacologic blockade pf NPRB with P19 [17, 18]. There was no statistically significant difference in apoptosis between control and KD cells treated with serum. These data are consistent with the notion that beneficial effects of NPRC KO on the apoptotic response was mediated, at least part, by activation of NPRB by CNP.

11. Figure 1G: The increased apoptosis is interesting and should be confirmed with additional techniques and complemented with mechanistic insight.

Serum deprivation has been utilized for several decades to induce cell death by the removal of pro-survival growth factors [19]. Moreover, our lab and others have used serum deprivation to induce apoptosis in podocytes [2, 20]. In response to your comment, the mechanisms causing cell death has expanded to include multiple distinct pathways [21]. In addition, there is overlap between of the pathways promoting cell death. For example, autophagy often progresses to apoptosis [21, 22] and if apoptotic

cells are not efficiently cleared, the cells undergo programed cell necrosis [21-23]. The methodology used in our study (annexin V staining) is commonly used to detect apoptosis and, given the complexity of investigating types of cells death and the current focus of this manuscript, we believe the studies are beyond the focus of this manuscript.

12. Figure 2: How is the protein level of NPR-C in glomeruli from both genotypes? Is NPR-C expression preserved in other types of cells? (was NPR-C expression in rest of kidney, after separation of glomeruli, preserved?)

As discussed in the methods section, the Cre mouse was obtained from Jackson labs (model No. 008205) and permits inducible expression of Cre recombinase specifically in podocytes. The podocyte specificity of the system was documented in the initial publication [24] and this model has been used in multiple manuscripts since the initial paper was published. Figure 3A&B and supplementary figure S2 demonstrate that expression of Cre recombinase under the regulation of the podocyte specific podocin promoter in tdTomato mice (see methods) causes expression of green fluorescence in a pattern consistent with expression of Cre recombinase specifically in podocytes. In mice containing 2 "floxed" alleles of NPRC, this same promoter system specifically deletes NPRC in podocytes. In addition, we demonstrated that levels of NPRC were significantly decreased in the glomerular preparations by both quantitative RT-PCR (figure 2C) and immunoblotting (figure 2D & 2E) despite expression of NPRC in multiple cell types in the kidney [1, 12, 25]. Taken together with a large literature suggesting specificity of this approach to KO of podocyte proteins, these data suggest that podocyte specific KO of NPRC was successful.

13. Figure 4: The y-axis is named number of mice with Glomerulosclerosis. The legend shows normal, mild, moderate and severe sclerosis. Please label the y-axis accordingly (staging of glomerulosclerosis, histology of glomeruli?). It would help understandings to present the data in %, as is described in corresponding results section. ("~86% of the WT controls have no glomerulosclerosis and ~14 % mild glomerulosclerosis"). The present Figure is totally unclear because it does not relate the "depicted numbers" to the total number of study mice. Figure 4D-F: The columns "Absent" and "Present" in the diagram are redundant. Please show only the column "Present".

The percentage of mice with glomerulosclerosis (GS) relates to number of mice with (absent) or without (Present) evidence of GS. For example, in Figure 4C, the TG wild type group has 3 mice without GS, 5 mice with mild GS, 2 mice with moderate GS and 11 mice with severe GS. The total number of mice is 21. As a result, there are 18 mice with GS (5+2+11) divided by 21 mice without GS, which is approximately ~85.7% of TG wild type mice with GS.

To simplify Figure 4, we have eliminated Figure 4C from the manuscript and reported the percentage of mice with or without GS; however, we have retained Figure 4C in the Supporting Data to provide the exact numbers of mice with mild, moderate and severe GS as reported by our pathologist (Dr. Buckley). We decided to present the graphs of

the histopathologic findings as the percentage of mice with the specified abnormality to permit a more effective comparison of the differences between the experimental groups in studies with an imbalance in the number of mice in each group; however, the statistical evaluation was performed using the number of mice with the specified histologic abnormality.

14. Figure 4E: Why did you interchange black/white in Fig. E?

We have corrected the black/white problem in labeling the percentages.

15. Figure 4E: Does podocyte apoptosis induce tubulointerstitial inflammation? Where is the link between these two observations?

Podocyte damage/cell death disrupts the glomerular filtration barrier and promotes high levels of proteinuria. As discussed in the text of the manuscript, the level of proteinuria may contribute to tubulointerstitial inflammation. Filtered proteins are resorbed by the proximal tubules and processed by lysosomes and endoplasmic reticulum [26]. In the setting of heavy proteinuria, these intracellular pathways are stressed [27], which results in the secretion of cytokines that attract and activate inflammatory cells [28] and produce profibrotic mediators such as TGF-beta [29].

16. Figure4E: Figure 4E must illustrate in clear way how inflammation was measured.

The pathologist evaluating the renal histopathology provided a detailed descript of the methodology for assessing tubulointerstitial inflammation. Please see the Methods Section for details.

17. Figure 5: The mRNA and protein results are very discrepant. The mRNA expression levels of alpha- SMA and Fibronectin are only slightly impacted by genotypes and conditions (Figure 5A-D). The Western blots instead show huge inductions in WT mice and almost full inhibition of this induction in the KO (Figures 5E-H). How do you explain this mismatch between the regulation of mRNA and protein levels?

We also noticed the discrepancy in the mRNA expression and protein levels in the glomerular preparations. Review of the literature suggests that fibronectin accumulates in the glomerulus through both local synthesis and by deposition from the circulation [30, 31]. This was demonstrated by a liver specific fibronectin knockout to decrease circulating fibronectin [31]. In these KO mice, there is a decrease in both mesangial expansion and fibronectin accumulation the kidneys of diabetic mice. In addition, the major source of fibronectin in early stages of FSGS is from the circulation with deposition of glomerular fibronectin occurring later in the disease process. We suspect that the discrepancy in the mRNA expression and protein levels is related to greater deposition of fibronectin from the circulation in damaged glomeruli compared to

glomeruli with less severe glomerular injury. This possibility has been mentioned in the discussion section.

18. As mentioned in the discussion, NPs inhibit the master regulator of fibrosis, TGF-beta, by phosphorylating SMADs on an inhibitory site. Did you analyze SMAD phosphorylation? Is the inhibitory phosphorylation increased in the KO mice? Do NPRC-deficient podocytes express less proinflammatory and profibrotic molecules?

TGF-beta activates its receptor, which phosphorylates Smad3 on Ser423 and Ser425 [32].. Phosphorylated Smad3 combines with Smad4 to form a Smad complex,which then translocates into the nucleus to initiate mRNA transcription associate with inflammation and collagen formation [32].. ANP stimulates cGMP generation and actives cGMP kinase 1 (cGK1), which then phosphorylates Smad3 on Ser309 and on Thr388, and inhibits the nuclear translocation of the Smad complex and , in turn, fibrosis [32]. 00042.2016). Despite an extensive search, we were unable to locate antibodies to phospho-SMAD3 Ser309 or phospho-SMAD3 Thr388.

There are antibodies for phosphor-Smad3 on Ser423 and Ser425, and we attempted to determine if these Smad phosphoryaltion sites were affected in our KO mice but were unable to detect a signal. It is difficult to interpret these results because exploring cell signaling in animal models is complicated by: 1. Changes in modifications to cell signaling molecules involved in the response (for example phosphorylation and dephosphorylation in vivo, and repeated freeze-thaw cycles of stored samples) and 2. The time point chosen to harvest tissues (late versus early in the fibrotic response). We did not screen for additional proinflammatory or profibrotic molecules.

20. **Proteinuria versus kidney function: Please discuss appropriately the discrepant results from Figures 3 (marked Albuminuria) and 7 Cystatin C levels barely affected)**

It is common to have a large disparity in proteinuria and renal function in both animals and humans, especially in the early stages of glomerular diseases. The amount of proteinuria is dependent on the integrity of the filtration barrier, and podocytes play a key role in maintaining permselectivity. In contrast, decreased renal function results from nephron loss, which typically occurs with ongoing kidney injury. As result, animal models and humans may have high levels of proteinuria but well-preserved renal function as measured by the glomerular filtration rate (GFR). In later stages of the disease, nephron loss causes a decrease in GFR.

21. **Breeding Scheme: It is difficult to understand how the diverse transgenic mouse models were generated. It could help to include a scheme of the mice breedings.**

A detailed breeding scheme is provided in the Figure S7.

REVIEWER #2:

1. The two parts of the study, in vitro and in vivo, are linked by the fact that the NPRC was genetically inactivated in both. Otherwise, the results of the in vitro studies are not taken up in the in vivo part. Does the local production of CNP in podocytes play also a role in vivo in terms of an autocrine/paracrine effect? Is CNP regulated in podocytes in the animal model used? Or are the observed protective effects of NPRC deletion due to an enhanced effect of ANP / BNP via the NPRA? It is understandable that this question is very difficult to answer experimentally, but it should at least be discussed in more detail.

The relative podocyte protective effects of ANP, BMP and CNP is an important consideration that was not adequately addressed in the initial manuscript. The review is also correct that it is difficult to determine the relative autocrine/paracrine protective effects each ANP, BNP and/or CNP in vivo. It is certainly possible that podocyte CNP has local autocrine effects as suggested in our paper, but CNP is produced by multiple cell types in the kidney [1, 25, 33] and paracrine effects of CNP are also a consideration. In addition, given the high levels of NPRA compared to NPRB in podocytes [1, 25], ANP and BNP signaling may play a dominant podocyte protective role. To address these possibilities, we have performed additional studies to assess the effects of ANP and CNP on both cGMP generation and apoptosis (Figures 2A & 2B). These studies suggest that activation of NPRA was more effective at inhibiting apoptosis compared to activation of NPRB by CNP using equivalent doses of ANP and CNP.

2. **Line 6, results: Reference no. 23 is given as a reference for the expression of NPRB in podocytes. This should be checked, as the paper mentioned deals with the expression of CNP in the kidney, but not with NPRB. Other studies cited as evidence for the expression of NPRB in podocytes also show very strong expression of NPRA (receptor for ANP and BNP) and NPRC, but at best very weak expression of NPRB in podocytes (references 14 and 16). On the contrary, a recent study using in situ hybridization showed the expression of NPRB in glomerular endothelial cells and mesangial cells, but not in podocytes (Heinl, ES et al.; Pflugers Archiv 2023, 475(3): 343-360). Taking into account the results of these studies, in particular the strongly dominant expression of NPRA in podocytes, the influence of NPRC knockdown on the effects of ANP or BNP in cultured podocytes (cGMP, apoptosis) should be investigated**.

The reviewer is correct the reference 23 does not refer to NPRB. This reference has been removed ss a reference for NPRB but is still used as a reference for CNP throughout the manuscript. We have also added reference 13, 14 and 16 as a reference for expression of NPRB.

With regard to NPRB expression in podocytes, previous studies from our lab and from Lewko et al [34] found responsiveness to CNP in cultured podocytes suggesting

expression of NPRB in this cell type. In addition, single cell sequencing studies have consistently found NPRB in mouse and human kidney although he levels NPRB expression in podocytes are quite low compared to NPRA [1, 13]. While we can only speciate, one possibility is that the lack of NPRB expression using in situ hybridization in the **Heinl, ES et al** paper is related to sensitivity of the methodology.

In addition, we have performed additional studies to evaluate the effects NPRA versus NPRB signaling as discussed in Query 2 above.

3**. Fig. 1A: CNP immunofluorescence staining, at least in the present PDF, is not of sufficient quality to detect co-staining in podocytes. The quality of the image should be improved. It is also noted that "CNP was widely expressed in multiple cell types in the kidney". Please show overview images that reflect this expression.**

We agree and have removed this figure from the manuscript since it is not critical to the data presented in this manuscript. Please see response to Reviewer #1 query #1 for details.

4. Fig. 1E: It is clearly shown that the effect of CNP on cGMP levels is significantly enhanced in NPRC knockdown cells compared to control cells. It is not clear from Fig. 1E whether CNP has a significant effect at all in control cells. Does cGMP increase in control cells under CNP or is the effect completely abolished in the presence of NPRC.

The review is correct that the response to CNP in control cells is not significant. We suspect that the modest and non-significant increase in cGMP levels in the control podocytes was the result of: 1. High levels of NPRC expressed in podocytes compared to other kidney cell types [1, 12, 13] and 2. High levels of neprilysin in podocytes [2, 14, 15]. As a result, CNP is rapidly degraded, which limited the cGMP response.

5. The results of the in vitro experiments are compatible with the conclusion that the endogenous production of CNP in podocytes has a protective effect and that this is mediated via the NPRB. To further substantiate this, the NPRB could be knocked down in the podocyte cell culture used, with and without simultaneous NPRC inactivation, and the effect on apoptosis investigated. Overall, it should at least be discussed that there are a variety of studies showing that the NPRC is not only a clearance receptor, but also a signaling receptor that can be activated by CNP (for example: Bubb KJ, et. al.; Circulation. 2019 ;139(13): 1612-1628.; Moyes AJ, Hobbs AJ. Int J
Mol Sci. 2019 ;20(9): 2281).

We have provided additional data to address: 1. The role of NPRB signaling in the apoptotic response, and 2. The potential podocyte protective effects of NPRC signaling. To address NPRB signaling we blocked NPRB signaling using NPRB antagonist (P19) in KD cells and found that inhibiting NPRB enhanced podocyte apoptosis. These data

are presented in figure 1F and discussed in the manuscript. We have also discussed the potential role of NPRC signaling in the Discussion Section.

6. Fig. 4: Please show absolute values and not percentages.

There is some discrepancy in presenting the data in Figure 4 (see concerns raised by Reviewer #1 query #13). After considering options, we chose to present the graphs of the histopathologic findings as the percentage of mice with the specified histologic abnormality to permit a more effective comparison of the differences between the experimental groups in studies with an imbalance in the number of mice in each group; however, the statistical evaluation was performed using the number of mice with the specified histologic abnormality. In addition, we have provided the numbers of mice with mild, moderate and severe GS as reported by our pathologist (Dr. Buckley) in the Supporting Data (Fig S6). .

7. Fig. 5G: The arrow for fibronectin does not point to the dominant band of the Western blot. Which is the correct band? The molecular weight or a standard should be shown.

Fibronectin is a high molecular weight glycoprotein with a molecular weight of 440 kD. Glycosylation slows migration of the protein and can cause the immunoblot to appear as a large band on the immunoblot depending on the degree of glycosylation.
We have attempted to position the arrow on the dominant band of the immunoblot.

8. First page of introduction, last line: It should read "NPRA, NPRB and NPRC" instead of "NPRC, NPRB and NPRC".

We have corrected this mistake.

1. Wilson PC, Wu H, Kirita Y, Uchimura K, Ledru N, Rennke HG, et al. The single-cell transcriptomic landscape of early human diabetic nephropathy. Proc Natl Acad Sci U S A. 2019;116(39):19619-25. Epub 2019/09/12. doi: 10.1073/pnas.1908706116. PubMed PMID: 31506348; PubMed Central PMCID: PMCPMC6765272.
2. Wang L, Tang Y, Buckley AF, Spurney RF. Blockade of the natriuretic peptide clearance receptor attenuates proteinuria in a mouse model of focal segmental glomerulosclerosis. Physiol Rep. 2021;9(21):e15095. doi: 10.14814/phy2.15095. PubMed PMID: 34755480; PubMed Central PMCID: PMCPMC8578888.
3. Suga S, Nakao K, Hosoda K, Mukoyama M, Ogawa Y, Shirakami G, et al. Receptor selectivity of natriuretic peptide family, atrial natriuretic peptide, brain natriuretic peptide, and C-type natriuretic peptide. Endocrinology. 1992;130(1):229-39. doi: 10.1210/endo.130.1.1309330. PubMed PMID: 1309330.
4. Bennett BD, Bennett GL, Vitangcol RV, Jewett JR, Burnier J, Henzel W, et al. Extracellular domain-IgG fusion proteins for three human natriuretic peptide receptors. Hormone pharmacology and application to solid phase screening of synthetic peptide antisera. J Biol Chem. 1991;266(34):23060-7. PubMed PMID: 1660465.

5. Potter LR, Abbey-Hosch S, Dickey DM. Natriuretic peptides, their receptors, and cyclic guanosine monophosphate-dependent signaling functions. Endocr Rev. 2006;27(1):47-72. doi: 10.1210/er.2005-0014. PubMed PMID: 16291870.
6. Lucas KA, Pitari GM, Kazerounian S, Ruiz-Stewart I, Park J, Schulz S, et al. Guanylyl cyclases and signaling by cyclic GMP. Pharmacol Rev. 2000;52(3):375-414. PubMed PMID: 10977868.
7. Maack T, Suzuki M, Almeida FA, Nussenzveig D, Scarborough RM, McEnroe GA, et al. Physiological role of silent receptors of atrial natriuretic factor. Science. 1987;238(4827):675-8. Epub 1987/10/30. doi: 10.1126/science.2823385. PubMed PMID: 2823385.
8. Matsukawa N, Grzesik WJ, Takahashi N, Pandey KN, Pang S, Yamauchi M, et al. The natriuretic peptide clearance receptor locally modulates the physiological effects of the natriuretic peptide system. Proc Natl Acad Sci U S A. 1999;96(13):7403-8. PubMed PMID: 10377427; PubMed Central PMCID: PMCPMC22098.
9. Chusho H, Tamura N, Ogawa Y, Yasoda A, Suda M, Miyazawa T, et al. Dwarfism and early death in mice lacking C-type natriuretic peptide. Proc Natl Acad Sci U S A. 2001;98(7):4016-21. Epub 20010320. doi: 10.1073/pnas.071389098. PubMed PMID: 11259675; PubMed Central PMCID: PMCPMC31171.
10. Tamura N, Doolittle LK, Hammer RE, Shelton JM, Richardson JA, Garbers DL. Critical roles of the guanylyl cyclase B receptor in endochondral ossification and development of female reproductive organs. Proc Natl Acad Sci U S A. 2004;101(49):17300-5. doi: 10.1073/pnas.0407894101. PubMed PMID: 15572448; PubMed Central PMCID: PMCPMC534612.
11. Tsuji T, Kunieda T. A loss-of-function mutation in natriuretic peptide receptor 2 (Npr2) gene is responsible for disproportionate dwarfism in cn/cn mouse. J Biol Chem. 2005;280(14):14288-92. Epub 20050218. doi: 10.1074/jbc.C500024200. PubMed PMID: 15722353.
12. Staffel J, Valletta D, Federlein A, Ehm K, Volkmann R, Fuchsl AM, et al. Natriuretic Peptide Receptor Guanylyl Cyclase-A in Podocytes is Renoprotective but Dispensable for Physiologic Renal Function. J Am Soc Nephrol. 2017;28(1):260-77. doi: 10.1681/ASN.2015070731. PubMed PMID: 27153922; PubMed Central PMCID: PMCPMC5198264.
13. Wu H, Malone AF, Donnelly EL, Kirita Y, Uchimura K, Ramakrishnan SM, et al. Single-Cell Transcriptomics of a Human Kidney Allograft Biopsy Specimen Defines a Diverse Inflammatory Response. J Am Soc Nephrol. 2018;29(8):2069-80. doi: 10.1681/ASN.2018020125. PubMed PMID: 29980650; PubMed Central PMCID: PMCPMC6065085.
14. Kubiak-Wlekly A, Perkowska-Ptasinska A, Olejniczak P, Rochowiak A, Kaczmarek E, Durlik M, et al. The Comparison of the Podocyte Expression of Synaptopodin, CR1 and Neprilysin in Human Glomerulonephritis: Could the Expression of CR1 be Clinically Relevant? Int J Biomed Sci. 2009;5(1):28-36. PubMed PMID: 23675111; PubMed Central PMCID: PMCPMC3614758.
15. Debiec H, Guigonis V, Mougenot B, Decobert F, Haymann JP, Bensman A, et al. Antenatal membranous glomerulonephritis due to anti-neutral endopeptidase antibodies.

N Engl J Med. 2002;346(26):2053-60. Epub 2002/06/28. doi: 10.1056/NEJMoa012895. PubMed PMID: 12087141.
16. Koller KJ, Lowe DG, Bennett GL, Minamino N, Kangawa K, Matsuo H, et al. Selective activation of the B natriuretic peptide receptor by C-type natriuretic peptide (CNP). Science. 1991;252(5002):120-3. doi: 10.1126/science.1672777. PubMed PMID: 1672777.
17. Deschenes J, Dupere C, McNicoll N, L'Heureux N, Auger F, Fournier A, et al. Development of a selective peptide antagonist for the human natriuretic peptide receptor-B. Peptides. 2005;26(3):517-24. doi: 10.1016/j.peptides.2004.10.017. PubMed PMID: 15652659.
18. Buttgereit J, Shanks J, Li D, Hao G, Athwal A, Langenickel TH, et al. C-type natriuretic peptide and natriuretic peptide receptor B signalling inhibits cardiac sympathetic neurotransmission and autonomic function. Cardiovasc Res. 2016;112(3):637-44. Epub 2016/08/09. doi: 10.1093/cvr/cvw184. PubMed PMID: 27496871; PubMed Central PMCID: PMCPMC5157132.
19. Collins MK, Perkins GR, Rodriguez-Tarduchy G, Nieto MA, Lopez-Rivas A. Growth factors as survival factors: regulation of apoptosis. Bioessays. 1994;16(2):133-8. doi: 10.1002/bies.950160210. PubMed PMID: 8147843.
20. Lane BM, Chryst-Stangl M, Wu G, Shalaby M, El Desoky S, Middleton CC, et al. Steroid-sensitive nephrotic syndrome candidate gene CLVS1 regulates podocyte oxidative stress and endocytosis. JCI Insight. 2022;7(2). Epub 20220125. doi: 10.1172/jci.insight.152102. PubMed PMID: 34874915; PubMed Central PMCID: PMCPMC9018043.
21. Shen S, Shao Y, Li C. Different types of cell death and their shift in shaping disease. Cell Death Discov. 2023;9(1):284. Epub 20230804. doi: 10.1038/s41420-023-01581-0. PubMed PMID: 37542066; PubMed Central PMCID: PMCPMC10403589.
22. Booth LA, Tavallai S, Hamed HA, Cruickshanks N, Dent P. The role of cell signalling in the crosstalk between autophagy and apoptosis. Cell Signal. 2014;26(3):549-55. Epub 20131202. doi: 10.1016/j.cellsig.2013.11.028. PubMed PMID: 24308968; PubMed Central PMCID: PMCPMC4054685.
23. Karantza-Wadsworth V, White E. Role of autophagy in breast cancer. Autophagy. 2007;3(6):610-3. Epub 20070814. doi: 10.4161/auto.4867. PubMed PMID: 17786023; PubMed Central PMCID: PMCPMC2859167.
24. Shigehara T, Zaragoza C, Kitiyakara C, Takahashi H, Lu H, Moeller M, et al. Inducible podocyte-specific gene expression in transgenic mice. J Am Soc Nephrol. 2003;14(8):1998-2003. Epub 2003/07/23. PubMed PMID: 12874453.
25. Park J, Shrestha R, Qiu C, Kondo A, Huang S, Werth M, et al. Single-cell transcriptomics of the mouse kidney reveals potential cellular targets of kidney disease. Science. 2018;360(6390):758-63. doi: 10.1126/science.aar2131. PubMed PMID: 29622724.
26. Sharma S, Smyth B. From Proteinuria to Fibrosis: An Update on Pathophysiology and Treatment Options. Kidney Blood Press Res. 2021;46(4):411-20. Epub 20210615. doi: 10.1159/000516911. PubMed PMID: 34130301.
27. Ruggenenti P, Perna A, Remuzzi G, Investigators GG. Retarding progression of chronic renal disease: the neglected issue of residual proteinuria. Kidney Int. 2003;63(6):2254-61. doi: 10.1046/j.1523-1755.2003.00033.x. PubMed PMID: 12753315.

28. Liu D, Wen Y, Tang TT, Lv LL, Tang RN, Liu H, et al. Megalin/Cubulin-Lysosome-mediated Albumin Reabsorption Is Involved in the Tubular Cell Activation of NLRP3 Inflammasome and Tubulointerstitial Inflammation. J Biol Chem. 2015;290(29):18018-28. Epub 20150529. doi: 10.1074/jbc.M115.662064. PubMed PMID: 26025362; PubMed Central PMCID: PMCPMC4505048.
29. Bottinger EP. TGF-beta in renal injury and disease. Semin Nephrol. 2007;27(3):309-20. doi: 10.1016/j.semnephrol.2007.02.009. PubMed PMID: 17533008.
30. Shui HA, Ka SM, Lin JC, Lee JH, Jin JS, Lin YF, et al. Fibronectin in blood invokes the development of focal segmental glomerulosclerosis in mouse model. Nephrol Dial Transplant. 2006;21(7):1794-802. doi: 10.1093/ndt/gfl113. PubMed PMID: 16574682.
31. Klemis V, Ghura H, Federico G, Wurfel C, Bentmann A, Gretz N, et al. Circulating fibronectin contributes to mesangial expansion in a murine model of type 1 diabetes. Kidney Int. 2017;91(6):1374-85. Epub 20170131. doi: 10.1016/j.kint.2016.12.006. PubMed PMID: 28159318.
32. Shen K, Johnson DW, Gobe GC. The role of cGMP and its signaling pathways in kidney disease. Am J Physiol Renal Physiol. 2016;311(4):F671-F81. doi: 10.1152/ajprenal.00042.2016. PubMed PMID: 27413196.
33. Cataliotti A, Giordano M, De Pascale E, Giordano G, Castellino P, Jougasaki M, et al. CNP production in the kidney and effects of protein intake restriction in nephrotic syndrome. Am J Physiol Renal Physiol. 2002;283(3):F464-72. doi: 10.1152/ajprenal.00372.2001. PubMed PMID: 12167597.
34. Lewko B, Endlich N, Kriz W, Stepinski J, Endlich K. C-type natriuretic peptide as a podocyte hormone and modulation of its cGMP production by glucose and mechanical stress. Kidney Int. 2004;66(3):1001-8. doi: 10.1111/j.1523-1755.2004.00848.x. PubMed PMID: 15327393.
