# Supplementary material for: Podocyte specific knockout of the natriuretic peptide clearance receptor is podocyte protective in focal segmental glomerulosclerosis
Source: PLoS One. 2025 Mar 10;20(3):e0319424. doi: 10.1371/journal.pone.0319424 (PMC11892885; doi:10.1371/journal.pone.0319424)
Supplement: Attachment — Submitted filename: PLOS Rebuttal Letter.docx [file pone.0319424.s003.docx]

**RESPONSE TO THE REVIEW**

**Comments to the authors:**

**1. If the authors have adequately addressed your comments raised in a previous round of review and you feel that this manuscript is now acceptable for publication, you may indicate that here to bypass the “Comments to the Author” section, enter your conflict of interest statement in the “Confidential to Editor” section, and submit your "Accept" recommendation**.

- Reviewer #2: No response
- We have responded to individual comments below.

**2. Is the manuscript technically sound, and do the data support the conclusions? The manuscript must describe a technically sound piece of scientific research with data that supports the conclusions. Experiments must have been conducted rigorously, with appropriate controls, replication, and sample sizes. The conclusions must be drawn appropriately based on the data presented.**

- Reviewer #2: Partly
- We have made changes in the manuscript as outlined below to address comments raised by the reviewers.

**3. Has the statistical analysis been performed appropriately and rigorously?**

- Reviewer #2: Yes.

**4. Have the authors made all data underlying the findings in their manuscript fully available?**

- Reviewer #2: Yes

**5. Is the manuscript presented in an intelligible fashion and written in standard English?**

- Reviewer #2: Yes

**Reviewer comments to the authors:**

**1. As suggested, experiments were carried out on immortalized podocytes to examine the relevance of CNP signaling via NPRB. For this purpose, the substance P19 was used (Figure 1G). The effect of P19 in KD cells is low and the significance is very limited with a test number of n=4. This could easily be corrected by additional experiments. Furthermore, the concentration of P19 used is not indicated in the manuscript. This is particularly important because the affinity of P19 for NPRA and NPRB does not differ greatly, and the greatest affinity is actually for NPRC. This information can be found in reference 26 and should at least be discussed with all its consequences. A knockdown of NPRB would be the better method here.**

We have performed the experiments recommended by the reviewers and, although we only used an “N” of 4, the data is statistically significant, even after correcting for multiple comparisons. We do apologize for not including the concentrations of P19 (250 nM) in the manuscript, and this data has been added to the Methods Section. As the reviewers mentioned, the affinity of P19 for NPRA and NPRB does not differ greatly, and the greatest affinity is actually for NPRC. This is an important consideration that we did not address in the revised manuscript. Based on the following reference (Peptides 26 (2005) 517–524), we suggest that binding of P19 to either NPRC or NPRA is unlikely to affect the results in the knockdown (KD) podocytes because: 1. NPRC is absent in the KD cells, 2. The dosage of P19 used in the studies (250 nM) effectively binds NPRB (Kd 15.4 nM) but binds minimally to NPRA (NPRA Kd 575 nM). In contrast to the KD cells, NPRC is expressed in the control cells, which could affect the apoptotic response by blocking natriuretic clearance and enhancing CNP levels. We posit that blockade of NPRC in the control cells by P19 (Fig. 1G) had a minimal effect on the results because P19 has high affinity for both NPRB (Kd ~15 nM) and NPRC (0.0134 nM) and, in turn, would effectively inhibit both NP receptors (NPRB and NPRC). In this scenario, the benefits of increasing CNP levels by blockade NPRC would be reduced by pharmacologic blockade of NPRB. In the current study, the net effect was no change in the apoptotic response (Fig. 1G).

NOTE: affinity in the P19 reference is expressed as pK units and can be converted to Kd as follows pK = −log KD).

**2. The experiments comparing the effects of ANP and CNP on cGMP and apoptosis are very helpful. They clearly show that ANP exerts a protective effect on podocytes even without simultaneous inactivation of NPRC. This fits very well with in vivo experiments by other groups. However, the finding that a protective effect of CNP is only present when NPRC is inactivated calls into question the in vivo relevance of this effect, since podocytes in vivo also express NPRC. This should be discussed.**

We agree that, under the conditions used in the experiments (cell culture), ANP is more effect than CNP at inhibiting podocyte apoptosis at equimolar concentrations of ANP and CNP (Figure 2D). Indeed, the podocyte protective actions of CNP are only seen in the NPRC KD cells. The current studies do not, however, address potential CNP secretion under in vivo conditions or changes in CNP secretion during stress. Moreover, there are multiple CNP secreting cells in kidney that may contribute to enhance CNP levels and “overcome” the negative regulatory effects of NPRC. However, based on the data presented in the manuscript, we have modified the statements about the autocrine protective actions of CNP in the text of the manuscript to acknowledge the lack of a podocyte protective effect in the cells expressing NPRC

**3. As in the first version of the manuscript, the references should be carefully checked. For example, references 14 and 16 are mentioned to prove that “CNP is widely expressed in multiple cell types in mouse and human kidneys including glomerular podocytes (results, first paragraph)”. This information cannot be found in the papers. There may be a misunderstanding because “CNP” is mentioned in the gene lists. However, this is the gene of a “cyclic nucleotide phoshodiesterase” and not CNP whose gene name is NPPC. In addition, the references are used as evidence for “for the formation of cGMP in podocytes by the NPRB” (results, first paragraph). This is also not correct. Please correct me if I am wrong.**

The references 14 and 16 are single cell RNA sequencing studies. To obtain results for specific mRNAs investigators need to query the transcriptomics databases that accompany the manuscripts.

Database for these results, are available at the following websites: 1. <http://humphreyslab.com/SingleCell/> and 2. <https://susztaklab.com/EH/park/>. I have also added these weblinks to each manuscript citing single cell RNA sequencing studies in the Reference Section. I am also requesting guidance how to reference these results in the manuscript.

**4. It should be briefly discussed that the blood pressure values show a very high variability. Although this can occasionally occur with automated tail cuff measurement and is therefore not very unusual, it significantly weakens the authors' conclusion that there are no blood pressure differences between the genotypes.**

The tail cuff blood pressure measurement did show variability in the results, which reduces chances of detecting a difference between groups. We can only speculate on the causes, but the major finding in the BP studies is the lack of a difference in the Gq mice compared to the Gq-KO mice. This is important because a difference in BP could affect the severity of the kidney disease. The comparison in these 2 groups (GQ and Gq-KO) found very similar BP levels suggesting that a difference in BP did not affect the in vivo findings in the study.

**5. Legend Figure 2B: “KO mice” should be corrected to “KD cells”**

We have corrected this mistake in the text of the manuscript.

**Additional issues**: PLOS authors have the option to publish the peer review history of their article (what does this mean?). If published, this will include your full peer review and any attached files.

I do not think is necessary to publish the full review process.

**Reference**s

1.	Kubiak-Wlekly A, Perkowska-Ptasinska A, Olejniczak P, Rochowiak A, Kaczmarek E, Durlik M, et al. The Comparison of the Podocyte Expression of Synaptopodin, CR1 and Neprilysin in Human Glomerulonephritis: Could the Expression of CR1 be Clinically Relevant? Int J Biomed Sci. 2009;5(1):28-36. PubMed PMID: 23675111; PubMed Central PMCID: PMCPMC3614758.

2.	Debiec H, Guigonis V, Mougenot B, Decobert F, Haymann JP, Bensman A, et al. Antenatal membranous glomerulonephritis due to anti-neutral endopeptidase antibodies. N Engl J Med. 2002;346(26):2053-60. Epub 2002/06/28. doi: 10.1056/NEJMoa012895. PubMed PMID: 12087141.
